# Supplementary figures and images for: Gene Regulatory Network of ETS Domain Transcription Factors in Different Stages of Glioma
Source: J Pers Med. 2021 Feb 17;11(2):138. doi: 10.3390/jpm11020138 (PMC7922321; doi:10.3390/jpm11020138)

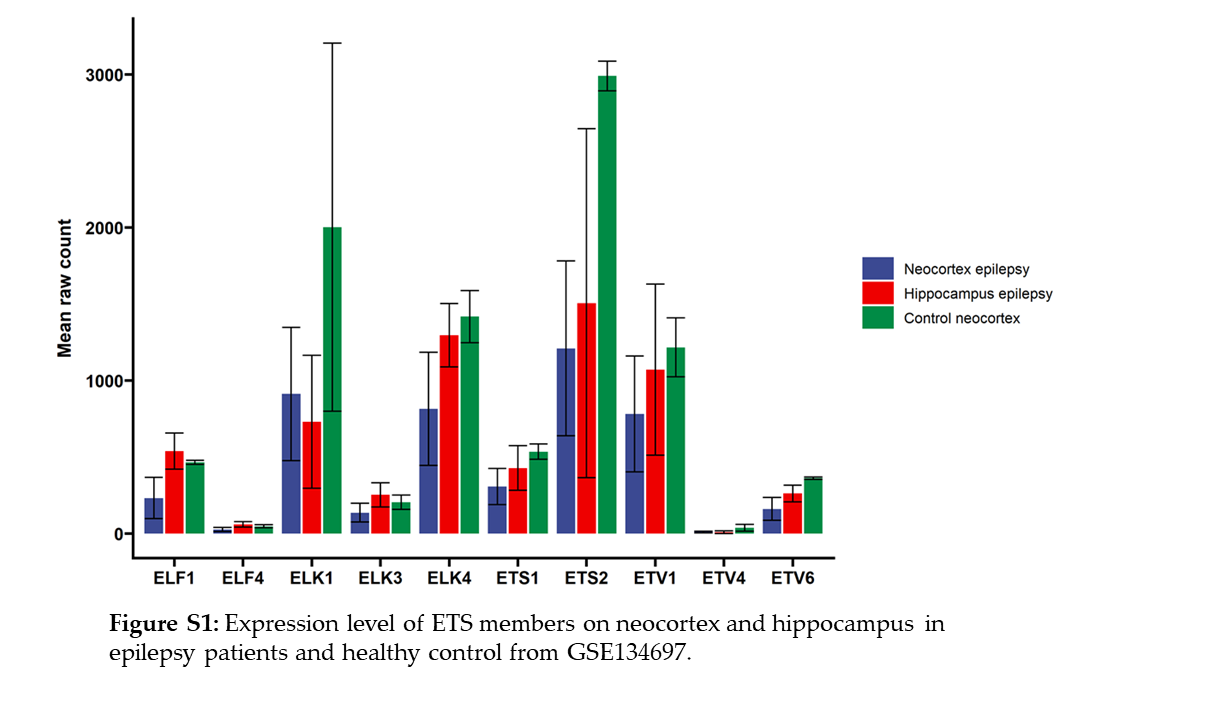

Supplement: Supplementary file 1 [file jpm-11-00138-s001.zip › SubFig1.tif]
